# Association Between Serum Growth Factors and Risk of Acute Exacerbation in Chronic Obstructive Pulmonary Disease: A One-Year Prospective Study

**DOI:** 10.3390/ijms262110584

**Published:** 2025-10-30

**Authors:** Hong-Yih Tien, Chung-Yu Chen, Chong-Jen Yu, Hao-Chien Wang

**Affiliations:** 1 Department of Emergency Medicine, National Taiwan University Hospital Yunlin Branch, Douliou City 640204, Yunlin County, Taiwan; 2 Department of Internal Medicine, National Taiwan University Hospital Yunlin Branch, Douliou City 640204, Yunlin County, Taiwan; 3 College of Medicine, National Taiwan University, Taipei 100225, Taiwan; 4 Division of Pulmonary and Critical Care Medicine, Department of Internal Medicine, National Taiwan University Hospital, Taipei 100225, Taiwan

**Keywords:** biomarkers, chronic obstructive pulmonary disease, fibroblast growth factor, growth factors, nerve growth factor, acute exacerbation, risk stratification

## Abstract

Chronic obstructive pulmonary disease (COPD) is characterized by persistent airflow limitation associated with enhanced chronic airway inflammation. Growth factors implicated in COPD’s inflammatory processes may serve as biomarkers for disease progression and exacerbation risk. This study evaluated the relationship between serum growth factors and COPD exacerbations over one year. Serum levels of eleven growth factors, including brain-derived neurotrophic factor (BDNF), leukemia inhibitory factor (LIF), fibroblast growth factor-2 (FGF-2), vascular endothelial growth factor (VEGF), nerve growth factor (NGF), epidermal growth factor (EGF), and stem cell factor (SCF), were measured in COPD patients at baseline. Participants were followed prospectively for one year, and associations between these biomarkers and acute exacerbations (AE) and frequent acute exacerbations (Frequent AE) were assessed using statistical analyses and receiver operating characteristic (ROC) curves. Among the study population, 42 patients experienced at least one AE within the follow-up period. Lower serum FGF-2 levels were significantly associated with increased AE risk (adjusted odds ratio significant after covariate adjustment). ROC analysis identified FGF-2 ≤ 9.12 pg/mL as a predictor of AE (AUC = 0.614, sensitivity = 64.3%, specificity = 57.1%, *p* = 0.032). For Frequent AE, eight patients experienced multiple exacerbations and exhibited significantly lower levels of NGF, EGF, FGF-2, and LIF. After adjustment, NGF remained significantly predictive; NGF ≤ 25.23 pg/mL demonstrated strong discriminatory power for Frequent AE (AUC = 0.797, *p* < 0.001). However, interpretations are limited by the small Frequent AE subgroup. Serum growth factors, particularly FGF-2 and NGF, are associated with COPD exacerbation risk. Lower serum FGF-2 may indicate a higher likelihood of acute exacerbations, while lower NGF strongly predicts frequent exacerbations. Larger studies and longer follow-ups are needed to confirm these biomarkers’ predictive utility.

## 1. Introduction

Chronic obstructive pulmonary disease (COPD) is a heterogeneous respiratory disease, and its global prevalence is projected to rise with ongoing exposure to risk factors and population aging [1]. COPD prevalence is higher among current and former smokers, individuals aged over 40, and males. Acute exacerbations of COPD significantly correlate with poor prognosis and reduced survival rates [2].

COPD is characterized by persistent and typically progressive airflow limitation due to chronic inflammatory responses in the lungs and airways triggered by inhalation of harmful particles or gases, particularly cigarette smoke [3]. Pathologically, COPD is characterized by expiratory airflow limitation resulting from chronic airway inflammation, loss of elastic recoil due to parenchymal destruction (emphysema), and narrowing of small airways [4]. Additional structural changes include collagen deposition in the bronchiolar wall, mucus plugging, and pulmonary vascular remodeling secondary to chronic hypoxia [5].

Diagnosis relies on pulmonary function testing, specifically a post-bronchodilator FEV_1_/FVC ratio below 0.7. The degree of airflow limitation is classified into four spirometric stages according to the GOLD 2007/2010 criteria, based on post-bronchodilator FEV_1_% predicted (Supplementary Figure S1). Separately, the overall severity of COPD in this study was assessed according to the GOLD 2017 classification, which incorporates symptom burden (e.g., CAT or mMRC scores) and the risk of exacerbations. Acute exacerbation (AE) refers to acute worsening of respiratory symptoms necessitating treatment changes. According to the 2017 GOLD report, AE involves sudden respiratory symptom deterioration beyond normal variations, requiring medication adjustments [6]. AE severity classifications include mild (symptom increase), moderate (requiring corticosteroids/antibiotics), and severe (emergency visits or hospitalizations). Frequent exacerbators experience two or more AEs annually [7].

Biomarkers, particularly serum biomarkers, are valuable indicators for assessing COPD severity and prognosis [8]. Recent studies have investigated both pulmonary and systemic inflammatory biomarkers in relation to COPD and its exacerbations. Airway-derived markers such as surfactant protein D (SP-D) and club cell protein 16 (CC16), as well as systemic markers including C-reactive protein (CRP) and fibrinogen, have been linked to disease activity and prognosis [9]. Moderate-quality evidence indicates that elevated levels of CRP, procalcitonin (PCT), interleukin-8 (IL-8), and tumor necrosis factor-α (TNF-α) are associated with bacterial infection in acute exacerbations of COPD (AECOPD) [10], while higher CRP concentrations predict an increased risk of hospital readmission [11]. Among these biomarkers, CC16, an anti-inflammatory protein secreted by bronchiolar club cells, is consistently reduced in COPD and correlates with airway epithelial injury, exacerbation frequency, and accelerated lung function decline [12]. Despite availability from other sources like exhaled condensates, induced sputum, or bronchoalveolar lavage (BAL), non-serum biomarkers are limited in clinical trials due to standardization and sampling challenges. Serum biomarkers, however, benefit from standardized collection protocols. Chronic lung injury often results in failed repair processes, leading to airway remodeling characterized by alveolar and small airway fibrosis. Cytokines and growth factors released during inflammation regulate airway structural changes, cellular proliferation, and collagen expression [13].

Recent investigations have increasingly examined the roles of growth factors in the pathogenesis and progression of COPD, emphasizing their contributions to airway remodeling, inflammation, and tissue repair. During episodes of severe pulmonary inflammation, growth factors released into the circulation may reflect systemic involvement and correlate with disease severity. This study examined 11 serum growth factors representing four pathobiological axes relevant to COPD: epithelial repair and mesenchymal remodeling (EGF, FGF-2, LIF); angiogenesis and tissue perfusion (VEGF, PDGF); neuro-immune signaling (NGF, BDNF); and hematopoietic/progenitor and tissue repair signalling (SCF, HGF). We limited the panel to 11 markers in order to (1) maintain a hypothesis-driven study design rather than a genome-wide screen (2) account for sample size constraints, and (3) minimize the multiple-testing burden to reduce the risk of inflated type I error.

Multiple growth factors have been implicated in the pathogenesis of COPD, contributing to inflammation, fibrosis, angiogenesis, and airway remodeling. Brain-Derived Neurotrophic Factor (BDNF) significantly contributes to fibrosis and emphysema in COPD [14,15]. Nerve Growth Factor-beta (NGF-β) enhances bronchial hyperresponsiveness [16,17,18]. Epidermal Growth Factor (EGF) influences cell proliferation and airway remodeling [19,20,21], while Hepatocyte Growth Factor (HGF) supports airway development and lung repair [22,23,24].

Leukemia Inhibitory Factor (LIF) can exacerbate airway hyperresponsiveness but may also provide anti-inflammatory benefits during pneumonia; however, its signaling mechanisms remain unclear [25,26]. Vascular Endothelial Growth Factor (VEGF), Platelet-Derived Growth Factor (PDGF), and Fibroblast Growth Factor (FGF) are crucial in angiogenesis, tissue repair, and epithelial regeneration [27,28,29,30,31,32,33]. Placental Growth Factor (PLGF) is involved in emphysema pathogenesis through apoptosis regulation [34,35]. Stem Cell Factor (SCF) promotes inflammatory responses and mast cell activation [36]. These growth factors play essential roles in COPD inflammation, fibrosis, angiogenesis, and airway remodeling. This one-year longitudinal study aims to examine correlations between serum growth factor concentrations and clinical COPD outcomes, evaluating their potential as biomarkers for monitoring acute exacerbations and frequent exacerbator phenotypes.

## 2. Results

### 2.1. Baseline Characteristics

A total of 138 patients with COPD were enrolled in this study. Of them, 23 patients withdrew, 1 died and 2 had missing values of growth markers, resulting in 112 patients eligible for analysis. Table 1 lists the baseline characteristics of these patients. Median age was 69 years with a range from 37 to 86 years. This cohort was predominantly male (98.2%). Sixty-two (55.4%) patients had quit smoking, 45 (40.2%) of them were current smokers and only 5 (4.5%) ones never smoked. The median FEV1 predicted value was 55% with a range from 18.5 to 107.6%.

The patients were further divided into with or without AE during the 1-year follow-up. Patients with subsequent AE had poor lung function at baseline in either the pulmonary function test or diffusing capacity of lung with carbon monoxide. No significant difference in the prevalence of comorbidities between the two groups was observed. However, patients with subsequent AE had higher neutrophils levels but had lower lymphocytes than those without. In addition, patients with subsequent AE tended to have lower triglyceride levels than those without (Supplementary Table S1). In addition, the baseline characteristics of patients by frequency of acute exacerbation (non-AE, AE and frequent AE [FAE] were detailed in Supplementary Table S2. We additionally examined the relationship between blood eosinophil count and 11 other growth factors, and found no significant associations (Supplementary Table S3).

### 2.2. Growth Markers and COPD AE During the 1-Year Follow-Up

The value of growth markers according to the presence of COPD AE during the 1-year follow-up is summarized (Table 2). The results showed that the FGF-2 level was significantly lower in patients with COPD AE compared to those without (4.5 vs. 12.4 pg/mL; *p* = 0.041), while the values of the remaining 10 growth markers did not significantly differ between the two groups (*p* > 0.05). In addition, the value of growth markers of patients by frequency of acute exacerbation (non-AE, AE and frequent AE [FAE] was present in Supplementary Table S4.

The multivariable logistic regression models showed that a higher level of FGF-2 was significantly associated with a lower risk of COPD AE with adjustment of covariates (odds ratio 0.972; 95% confidence interval [CI] 0.949–0.997) (Table 3). On the other hand, a higher level of SCF was significantly associated with a higher risk of COPD AE with adjustment of covariates (odds ratio 1.036; 95% CI 1.001–1.072). However, the remaining 9 growth markers were not significantly associated with the risk of being frequent AE in either the univariate analyses or multivariable analysis.

**Figure 1:**
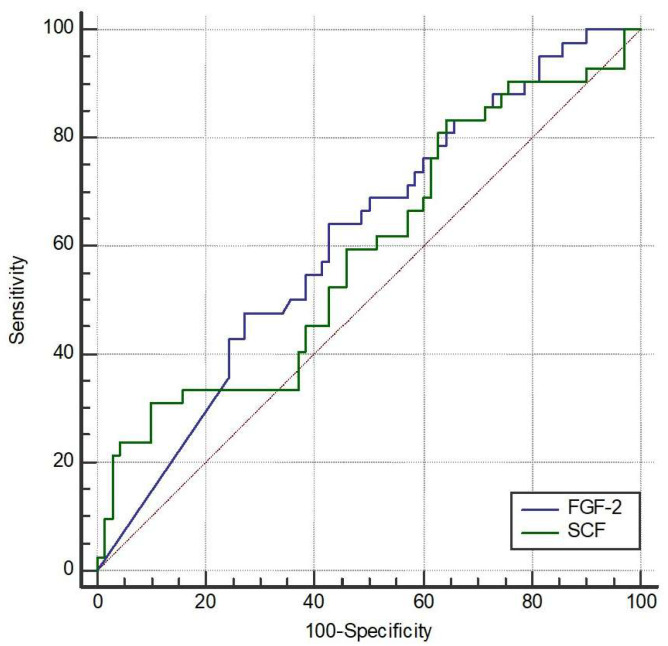
Receiver operating characteristic (ROC) curves of fibroblast growth Factor-2 (FGF-2) and stem cell factor (SCF) to discriminate the occurrence of acute exacerbation (AE). The area under the curve (AUC) for FGF-2 was 61.4% (95% CI 51.8–70.5%, *p* = 0.032). The optimal cutoff point for FGF-2 was determined to be 9.12 pg/mL, providing a sensitivity of 64.3% (95% CI 48.0–78.4%) and a specificity of 57.1% (95% CI 44.7–68.9%). The AUC for SCF was 58.9% (95% CI 49.2–68.1%, *p* = 0.117), and an optimal cutoff point was not determined due to lack of statistical significance.

We further explored the possible cutoffs of FGF-2 and SCF to discriminate the occurrence of AE. The area under the curve (AUC) of FGF-2 was 61.4% (95% CI 51.8% to 70.5%, *p* = 0.032) (Figure 1). The optimal cutoff of FGF-2 was 9.12 pg/mL with a sensitivity of 64.3% (95% CI 48.0% to 78.4%) and a specificity of 57.1% (95% CI 44.7% to 68.9%). The AUC of SCF was 58.9% (95% CI 49.2% to 68.1%, *p* = 0.117). The optimal cutoff of SCF was not available due to the non-significant ROC analysis.

### 2.3. Growth Markers and COPD AE Frequency During the 1-Year Follow-Up

We additionally conducted analysis of frequent AE which was defined as number of COPD AE ≥ 2 times. The result showed that the NGF, EGF, FGF-2, and LIF levels were significantly lower in patients with frequent AE than those without (*p* < 0.05) (Table 4). However, the values of the remaining 7 growth markers were not significantly different between patients with frequent AE and those without (*p* > 0.05).

The multivariable model showed that a higher level of NGF was significantly associated with a lower risk of frequent AE with adjustment of covariates (odds ratio 0.93; 95% CI 0.87–0.99) (Table 5). However, the remaining 10 growth markers were not significantly associated with the risk of being frequent AE in either the univariate analyses or multivariable analysis.

**Figure 2:**
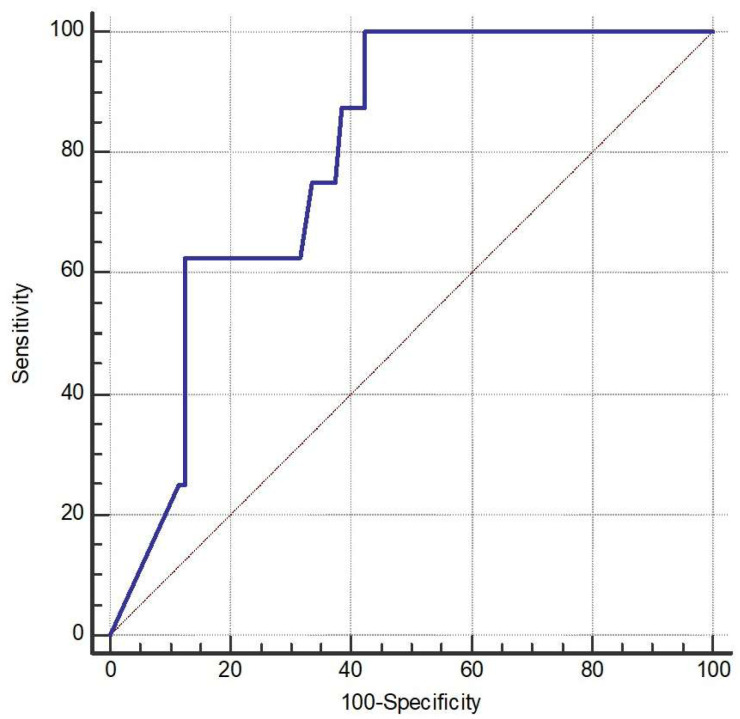
Receiver operating characteristic (ROC) curve (the blue line) of nerve growth factor (NGF) for discriminating the occurrence of acute exacerbation (AE). The AUC for NGF was 79.7% (95% CI 71.1–86.8%, *p* < 0.001). The optimal cutoff point for NGF was identified at 25.23 pg/mL, yielding a sensitivity of 100% (95% CI 63.1–100%) and a specificity of 57.7% (95% CI 47.6–67.3%).

The AUC of NGF was 79.7% (95% CI 71.1% to 86.8%, *p* < 0.001) (Figure 2). The optimal cutoff of NGF was 25.23 pg/mL with a sensitivity of 100% (95% CI 63.1% to 100%) and a specificity of 57.7% (95% CI 47.6% to 67.3%).

Statistical analysis revealed that patients who experienced acute exacerbations (AEs) during the one-year follow-up had significantly lower serum levels of fibroblast growth factor-2 (FGF-2), indicating that higher FGF-2 levels were associated with a reduced risk of exacerbation. After adjusting for covariates, both FGF-2 and stem cell factor (SCF) remained statistically associated with acute exacerbation.

Receiver operating characteristic (ROC) curve analysis was used to assess the ability of FGF-2 and SCF to discriminate between patients with and without acute exacerbations after one year. The area under the curve (AUC) for FGF-2 was 0.614, with a Youden index cutoff value of ≤9.12, yielding a sensitivity of 64.3% and specificity of 57.1%**.** Although the discriminative power was modest and the diagnostic utility limited, the result reached statistical significance (*p* = 0.032). In contrast, the AUC for SCF was 0.589, which was not statistically significant (*p* = 0.117); therefore, no optimal cutoff point was determined for SCF.

For the prediction of frequent acute exacerbations (frequent AEs), eight patients met the criteria for frequent exacerbations. These patients exhibited significantly lower levels of NGF, EGF, FGF-2, and LIF (*p* < 0.05). However, after adjusting for covariates, only NGF remained significantly associated with frequent exacerbations, with higher NGF levels indicating a lower risk.

**Figure 3:**
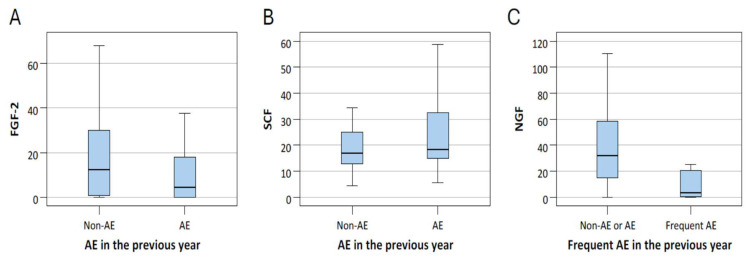
The level of FGF-2 (**A**) and SCF (**B**) of patients with and without acute exacerbation (AE), and NGF (**C**) levels of patients with and without frequent AE. The center line within the box represents the median, while the lower and upper edges of the box indicate the 25th and 75th percentiles, respectively.

ROC analysis showed that NGF had an AUC of 0.797 (*p* < 0.001), indicating acceptable to near-excellent discriminative power. The optimal cutoff value determined by the Youden index was ≤25.23. Nevertheless, due to the small number of frequent exacerbators (*n* = 8), these findings should be interpreted with caution. At last, we provided box plots of the three biomarkers that remained significant (FGF-2, NGF, and SCF) to illustrate how their levels differed between patients with and without exacerbations (Figure 3).

## 3. Discussion

This study explored the roles of fibroblast growth factor (FGF) and nerve growth factor (NGF) in chronic obstructive pulmonary disease (COPD) exacerbations, highlighting significant findings with potential clinical implications. FGF-1 and FGF-2, binding to their receptor FGFR-1, indeed promote airway remodeling by increasing granulation tissue formation, stimulating fibroblast proliferation, enhancing collagen synthesis, and promoting angiogenesis. This FGF/FGFR signaling pathway is critical for tissue repair and development, and its activation leads to various cellular responses, including enhanced cell growth, differentiation, and new blood vessel formation [37,38,39]. FGF2 plays an important role in asthma and COPD, as it is involved not only in the regulation of inflammatory cells but also in mediating interactions between immune cells and airway structural cells. The potential role of FGF2 in regulating inflammation suggests that it could serve as a therapeutic target for chronic inflammatory diseases [40,41,42,43,44].

A major finding of this study is the significant association between lower serum FGF-2 levels and increased risk of acute exacerbations, suggesting its potential as a biomarker to predict COPD exacerbation risk. This aligns with previous studies indicating that FGF-2 can reduce inflammatory cell infiltration and alveolar destruction, potentially attenuating COPD progression [45,46,47,48]. However, conflicting data also suggest that FGFs may promote fibrosis under certain conditions, highlighting their dual and context-dependent roles [49]. Importantly, smoking negatively correlates with serum FGF-2 levels, underscoring a potential mechanism linking smoking and increased COPD exacerbation risk [50,51].

Another critical finding concerns NGF, a neurotrophic factor involved in regulating autonomic neurons and immune responses, particularly in allergic and inflammatory airway conditions [52,53,54]. Elevated NGF correlates with increased airway hyperresponsiveness and remodeling [55,56], while cigarette smoke exposure, specifically nicotine, increases NGF expression in lung tissue [57]. Notably, this study found that lower NGF levels significantly predicted frequent COPD exacerbations. The identified NGF cutoff level (≤25.23 pg/mL) showed high discriminatory power for frequent exacerbations, highlighting its promising role as a predictive biomarker. However, these findings contrast with previous studies reporting elevated NGF in severe COPD, possibly due to extensive lung tissue damage in advanced stages, warranting further investigation [58,59].

Recent studies have highlighted the role of growth factors in chronic obstructive pulmonary disease (COPD). Preclinical research has shown that mesenchymal stem cells (MSCs), owing to their anti-inflammatory, reparative, and immunomodulatory properties, may represent a promising new therapeutic strategy for COPD [60]. Clinical evidence further indicates that serum fibroblast growth factor-2 (FGF-2) and vascular endothelial growth factor (VEGF) levels are significantly elevated during acute exacerbations, reflecting heightened disease activity and severity [61,62]. Conversely, reduced serum brain-derived neurotrophic factor (BDNF) levels observed in AECOPD patients compared with healthy controls suggest impaired neurotrophic regulation and potential neuronal involvement in disease progression [63].

In large cohorts such as COPDGene and ECLIPSE [64], higher blood eosinophil counts have been associated with an increased risk of exacerbation in COPD. However, in our study, no significant correlation was observed between eosinophil count and FGF-2 or NGF levels. This discrepancy may be attributed to several factors. First, our study had a smaller sample size and a single-center design, which may have limited the statistical power to detect subtle associations. Second, eosinophil count mainly reflects Th2-mediated airway inflammation, whereas FGF-2 and NGF are involved in airway remodeling, angiogenesis, and neurogenic inflammation, reflecting distinct biological pathways. Finally, population heterogeneity and differences in inflammatory endotypes may have contributed to the lack of association. These findings suggest that FGF-2 and NGF represent alternative biological dimensions of COPD beyond eosinophilic inflammation.

Beyond growth factors, other classes of circulating biomarkers have recently been investigated to capture systemic inflammation and cellular stress in COPD. For example, the ECLIPSE cohort further revealed that higher plasma cf-mtDNA levels predict future exacerbation rates in COPD, supporting its role as a potential biomarker of disease activity [65]. Since cf-mtDNA acts as a proinflammatory molecule involved in COPD-related hyperinflammation, this finding provides additional insight into the inflammatory pathways underlying disease exacerbation [66]. Although cf-mtDNA represents a different biological pathway from the growth factor signaling examined in our study, both types of biomarkers collectively highlight the multifaceted nature of COPD pathogenesis, encompassing inflammatory, structural, and metabolic dysregulation.

Several limitations of this study warrant discussion. Firstly, the complex inflammatory mechanisms of COPD were only partially captured, as the analysis did not include comprehensive inflammatory biomarkers from sputum or bronchoalveolar lavage fluid. Secondly, the absence of control groups comprising healthy individuals or smokers without COPD limits the specificity assessment of growth factor changes. Additionally, short-term follow-up and a relatively small sample size, particularly in the frequent exacerbation subgroup, necessitate cautious interpretation of the results. Future research with larger cohorts, longer follow-up durations, and comprehensive inflammatory assessments will be crucial for validating these biomarkers’ predictive utility and clinical relevance.

## 4. Materials and Methods

### 4.1. Data Collection of Participants

This was a prospective observational study conducted from 1 January 2012 to 31 December 2014. This study was conducted in accordance with the principles of the Declaration of Helsinki. The study protocol was reviewed and approved by the Institutional Review Board of National Taiwan University Hospital. Written informed consent was obtained from all participants prior to study enrollment. A total of 138 outpatients aged 40 to 99 years with chronic obstructive pulmonary disease (COPD) were enrolled. The diagnosis of COPD was based on the GOLD criteria, defined as a post-bronchodilator FEV_1_/FVC ratio of less than 70% and a post-bronchodilator FEV_1_ of less than 80% predicted. The severity of COPD was classified into four groups (A–D) based on the GOLD 2017 classification, which incorporates both symptom burden and exacerbation risk. Because this study was exploratory rather than a confirmatory trial, we did not perform a priori sample size calculation or power analysis.

Inclusion criteria were (1) age between 40 and 100 years; (2) diagnosis of COPD confirmed by spirometry. Exclusion criteria included age under 40 years; recent treatment for acute exacerbation (AE) within 30 days (hospitalization, antibiotic or corticosteroid adjustment); hospitalization for non-COPD pulmonary diseases (e.g., pneumonia) within 6 weeks; diagnosis of asthma; other pulmonary diseases (e.g., pneumoconiosis, tuberculosis with extensive pulmonary destruction, bronchiectasis, cystic fibrosis, interstitial lung disease); or terminal cancer with a life expectancy of less than five years.

Each participant underwent a single pulmonary function test and completed symptom assessments using the Medical Research Council (MRC) scale, COPD Assessment Test (CAT), and symptom diary. Serum samples were collected for growth factor analysis. Among the enrolled patients, 23 withdrew from the study, 1 patient died, and 2 samples were incomplete. In other words, the 26 excluded patients had no outcome data available during the follow-up period. Ultimately, 112 patients were included in the final analysis. Patients were grouped based on the occurrence of acute exacerbation (AE) or frequent exacerbations (defined as ≥2 AEs) during the one-year follow-up period.

Collected data included demographic information (sex, age, height, and weight), smoking and alcohol consumption history, personal and family medical history, medication use, physical and imaging examinations (chest radiography and chest computed tomography when indicated), pulmonary function parameters (FVC%, FEV_1_%, FEV_1_/FVC), and laboratory tests including complete blood count (CBC), erythrocyte sedimentation rate (ESR), liver and renal function tests, and arterial blood gas analysis.

### 4.2. Biomarker Detection

Quantification of serum growth factors was performed using a multiplex immunoassay technique (Immunoassays: Growth Factor 11-plex Human ProcartaPlex Panel), which offers advantages of high throughput, accuracy, and sensitivity. This method allows for the simultaneous quantification of multiple cytokines and growth factors from a small sample volume (25–50 μL), making it suitable for analysis across various sample types.

This study aimed to investigate the predictive potential of serum growth factor concentrations, in combination with clinical indicators, for acute and frequent exacerbations in patients with COPD over a one-year follow-up period. The goal was to evaluate the feasibility of utilizing these biomarkers for clinical risk stratification, to facilitate early therapeutic intervention, reduce recurrence and hospitalization rates, and ultimately improve overall quality of care for COPD patients.

### 4.3. Statistical Methods

Due to most of the growth markers were not normally distributed, non-parametric statistics were used in this study. The baseline characteristics and growth markers of patients with and without acute exacerbation (AE) during the 1-year follow-up were compared using the Mann–Whitney U test for continuous variables or Fisher’s exact test for categorical variables. The association between growth markers and binary outcomes (i.e., AE or frequent AE during the 1-year follow-up) was investigated by univariate and multivariable logistic regression analyses in which the later was adjusted for age, sex, body mass index and smoking status. Finally, the performance of growth markers on discriminating AE or frequent AE was evaluated by the receiver operating characteristic (ROC) curve analysis. Statistics analyses were conducted using IBM SPSS 25 (IBM SPSS Inc., Chicago, IL, USA). Significance was set at 0.05 with two-side test. No adjustment of multiple testing was made in this study.

## 5. Conclusions

Growth factors and their receptors play complex roles in COPD pathogenesis, involving processes such as airway remodeling, epithelial and endothelial cell apoptosis, and pulmonary vascular remodeling. This complexity suggests that targeting growth factors therapeutically may yield unpredictable and varying outcomes among individuals [67]. Our study revealed significantly lower serum fibroblast growth factor-2 (FGF-2) levels in COPD patients who experienced acute exacerbations compared to those who did not, even after adjusting for covariates. An optimal predictive cutoff value for acute exacerbation was identified as ≤9.12 pg/mL. Similarly, higher nerve growth factor (NGF) levels were significantly associated with reduced risk of frequent exacerbations, with an optimal cutoff of ≤25.23 pg/mL. However, caution is advised due to the limited sample size (only eight patients) in the frequent exacerbation subgroup, highlighting the need for validation in larger cohorts.

Our findings suggest that incorporating multiple growth factors into predictive models could enhance sensitivity for forecasting acute and frequent exacerbations in COPD. Future research should include longitudinal sampling during and after exacerbations to account for medication effects and biomarker dynamics. Expanding sample sizes, employing diverse sample types such as sputum, bronchoalveolar lavage, or lung tissue biopsies, and leveraging big data analytics could further refine predictive models [68]. Ultimately, validated biomarkers could significantly improve early detection, prevention, and individualized management strategies in COPD, potentially reducing healthcare costs and mortality and enhancing patient quality of life.

## Figures and Tables

**Table 1:** Selected baseline characteristics of the study participants with chronic obstructive pulmonary disease * stratified by the presence of acute exacerbation during the 1-year follow-up.

Variable	Total (*n* = 112)	Non-AE (*n* = 70)	AE (*n* = 42)	*p*
Demographics				
Male, *n* (%)	110 (98.2)	69 (98.6)	41 (97.6)	1.000
Age, years	69 (63, 75)	69 (62, 74)	69 (64, 75)	0.371
BMI, kg/m^2^	23.6 (21.6, 25.7)	23.7 (22.3, 25.7)	22.4 (20.9, 25.6)	0.115
Smoking, *n* (%)				1.000
Never	5 (4.5)	3 (4.3)	2 (4.8)	
Current	45 (40.2)	28 (40.0)	17 (40.5)	
Quit	62 (55.4)	39 (55.7)	23 (54.8)	
GOLD classification, *n* (%)				0.143
1	13 (11.6)	11 (15.7)	2 (4.8)	
2	53 (47.3)	35 (50.0)	18 (42.9)	
3	39 (34.8)	21 (30.0)	18 (42.9)	
4	7 (6.3)	3 (4.3)	4 (9.5)	
GROUP classification, *n* (%)				0.166
A	46 (41.1)	34 (48.6)	12 (28.6)	
B	23 (20.5)	14 (20.0)	9 (21.4)	
C	25 (22.3)	13 (18.6)	12 (28.6)	
D	18 (16.1)	9 (12.9)	9 (21.4)	
Lung function				
FVC, L	2.6 (2.0, 3.3)	2.9 (2.2, 3.3)	2.3 (1.9, 3.0)	0.006
FVC predicted, %	68 (55, 80)	70 (60, 89)	65 (48, 73)	0.031
FEV1, L	1.3 (1.1, 1.7)	1.4 (1.2, 1.8)	1.2 (0.8, 1.6)	0.010
FEV1 predicted, %	55 (43, 69)	57 (46, 72)	49 (36, 58)	0.004
FEV1/FVC, %	53 (44, 61)	55 (44, 62)	51 (42, 61)	0.298
Comorbidity				
Cardiovascular disease	9 (8.0)	6 (8.6)	3 (7.1)	1.000
Cerebrovascular disease	19 (17.0)	11 (15.7)	8 (19.0)	0.795
Peptic ulcer disease	19 (17.0)	13 (18.6)	6 (14.3)	0.613
Liver disease	5 (4.5)	4 (5.7)	1 (2.4)	0.649
Diabetes	13 (11.6)	9 (12.9)	4 (9.5)	0.764
Renal disease	5 (4.5)	4 (5.7)	1 (2.4)	0.649
Malignancy	8 (7.1)	3 (4.3)	5 (11.9)	0.149
CCI total score (excluding pulmonary disease)	1.0 (1.0, 2.0)	1.0 (1.0, 2.0)	1.0 (1.0, 3.0)	0.801

* The control group (healthy individuals, smokers or non-smokers without COPD) was not considered for the analysis. FVC, forced vital capacity; FEV1, forced expiratory volume in one second; DLCO, diffusing capacity of lung with carbon monoxide; DL, diffusing capacity of lung; VA, alveolar volume; CCI, Charlson Comorbidity Index score; LDL, low density lipoprotein; HDL, high density lipoprotein; SGOT, serum glutamic-oxalocetic transaminase; CRP, C-reactive protein; Data were presented as frequency with percentage or median (interquartile range).

**Table 2:** The value of growth markers according to the presence of acute exacerbation during the 1-year follow-up.

Variable, pg/mL	Non-AE (*n* = 70)	AE (*n* = 42)	*p* Value §
NGF	32.5 (16.1, 61.4)	21.1 (3.7, 55.4)	0.154
BDNF	97.0 (65.7, 174.5)	95.2 (56.9, 138.5)	0.536
EGF	29.5 (19.0, 47.6)	24.9 (15.7, 34.5)	0.100
FGF-2	12.4 (0.8, 30.0)	4.5 (0.0, 18.1)	0.041
HGF	172.9 (151.4, 244.1)	210.1 (155.1, 256.3)	0.274
LIF	3.1 (2.4, 4.7)	2.5 (2.0, 3.6)	0.056
PDGF	255.0 (164.8, 431.3)	273.7 (156.9, 378.2)	0.829
PLGF	11.0 (6.4, 18.8)	9.7 (5.7, 15.8)	0.377
SCF	16.9 (13.0, 25.0)	18.5 (14.9, 32.5)	0.115
VEGF-A	82.8 (53.6, 136.2)	83.0 (55.4, 125.6)	0.983
VEGF-D	8.1 (5.7, 14.7)	8.6 (4.2, 13.0)	0.414

Abbreviation: AE, acute exacerbation; NGF, nerve growth factor; BDNF, brain-derived neurotrophic factor; EGF, epidermal growth factor; FGF-2, fibroblast growth Factor-2; HGF, hepatocyte growth factor; LIF, leukemia inhibitory factor; PDGF, platelet-derived growth factor; PLGF, placental growth factor; SCF, stem cell factor; VEGF-A, vascular endothelial growth factor-A; VEGF-D, vascular endothelial growth factor-D; Data were presented as median (interquartile range); § Mann–Whitney U test.

**Table 3:** Association between growth markers and risk of acute exacerbation during the 1-year follow-up.

	Univariate Analysis		Multivariable Analysis #	
Variable, pg/mL	OR (95% CI)	*p*	OR (95% CI)	*p*
NGF	1.00 (0.98–1.01)	0.427	1.00 (0.98–1.01)	0.513
BDNF	0.999 (0.995–1.003)	0.533	0.998 (0.994–1.003)	0.444
EGF	0.981 (0.961–1.002)	0.074	0.980 (0.959–1.001)	0.058
FGF-2	0.974 (0.951–0.997)	0.029	0.972 (0.949–0.997)	0.027
HGF	1.002 (0.997–1.006)	0.417	1.002 (0.997–1.007)	0.414
LIF	1.02 (0.98–1.05)	0.402	1.02 (0.98–1.05)	0.409
PDGF	0.999 (0.997–1.001)	0.380	0.999 (0.997–1.001)	0.324
PLGF	1.00 (0.96–1.04)	0.960	1.00 (0.96–1.04)	0.849
SCF	1.034 (1.001–1.068)	0.041	1.036 (1.001–1.072)	0.047
VEGF-A	1.00 (0.99–1.01)	0.950	1.00 (0.99–1.01)	0.855
VEGF-D	0.98 (0.96–1.01)	0.226	0.98 (0.96–1.01)	0.203

Abbreviation: OR, odds ratio; CI, confidence interval; NGF, nerve growth factor; BDNF, brain-derived neurotrophic factor; EGF, epidermal growth factor; FGF-2, fibroblast growth Factor-2; HGF, hepatocyte growth factor; LIF, leukemia inhibitory factor; PDGF, platelet-derived growth factor; PLGF, placental growth factor; SCF, stem cell factor; VEGF-A, vascular endothelial growth factor-A; VEGF-D, vascular endothelial growth factor-D; # Adjusted for age, sex, body mass index and smoking status.

**Table 4:** The value of markers according to frequent AE during the 1-year follow-up.

Variable, pg/mL	Infrequent AE (*n* = 104)	Frequent AE (*n* = 8)	*p* Value §
NGF	32.2 (15.0, 58.5)	3.7 (0.7, 20.4)	0.005
BDNF	97.0 (64.6, 156.8)	88.6 (51.8, 154.7)	0.701
EGF	28.4 (18.8, 44.4)	17.1 (14.3, 22.8)	0.025
FGF-2	10.9 (0.0, 26.7)	0.1 (0.0, 4.3)	0.021
HGF	182.2 (152.0, 243.3)	241.4 (174.6, 298.4)	0.175
LIF	3.0 (2.3, 4.5)	1.9 (1.6, 3.1)	0.047
PDGF	263.5 (166.6, 427.0)	235.0 (116.6, 359.7)	0.491
PLGF	10.7 (6.4, 17.9)	7.8 (3.6, 19.2)	0.483
SCF	17.0 (13.4, 26.1)	18.9 (12.1, 28.8)	0.752
VEGF-A	83.4 (55.5, 136.1)	52.8 (40.0, 108.2)	0.148
VEGF-D	8.4 (5.7, 14.3)	5.9 (4.1, 9.6)	0.175

Abbreviation: AE, acute exacerbation; NGF, nerve growth factor; BDNF, brain-derived neurotrophic factor; EGF, epidermal growth factor; FGF-2, fibroblast growth Factor-2; HGF, hepatocyte growth factor; LIF, leukemia inhibitory factor; PDGF, platelet-derived growth factor; PLGF, placental growth factor; SCF, stem cell factor; VEGF-A, vascular endothelial growth factor-A; VEGF-D, vascular endothelial growth factor-D; Data were presented as median (interquartile range); § Mann–Whitney U test.

**Table 5:** Association between growth markers and risk of frequent AE during the 1-year follow-up.

	Univariate Analysis		Multivariable Analysis #	
Variable, pg/mL	OR (95% CI)	*p*	OR (95% CI)	*p*
NGF	0.93 (0.88–0.99)	0.020	0.93 (0.87–0.99)	0.023
BDNF	1.00 (0.99–1.01)	0.538	1.00 (0.99–1.01)	0.604
EGF	0.93 (0.87–1.001)	0.055	0.93 (0.87–1.001)	0.061
FGF-2	0.88 (0.75–1.02)	0.090	0.87 (0.74–1.02)	0.086
HGF	1.004 (0.998–1.011)	0.197	1.005 (0.997–1.012)	0.209
LIF	0.53 (0.25–1.13)	0.100	0.50 (0.22–1.14)	0.100
PDGF	0.998 (0.994–1.002)	0.412	0.998 (0.994–1.003)	0.420
PLGF	0.98 (0.90–1.06)	0.577	0.98 (0.90–1.07)	0.617
SCF	1.01 (0.96–1.07)	0.626	1.01 (0.96–1.07)	0.668
VEGF-A	0.99 (0.98–1.01)	0.322	0.99 (0.98–1.01)	0.320
VEGF-D	0.94 (0.84–1.06)	0.331	0.94 (0.84–1.06)	0.334

Abbreviation: OR, odds ratio; CI, confidence interval; NGF, nerve growth factor; BDNF, brain-derived neurotrophic factor; EGF, epidermal growth factor; FGF-2, fibroblast growth Factor-2; HGF, hepatocyte growth factor; LIF, leukemia inhibitory factor; PDGF, platelet-derived growth factor; PLGF, placental growth factor; SCF, stem cell factor; VEGF-A, vascular endothelial growth factor-A; VEGF-D, vascular endothelial growth factor-D; # Adjusted for age, sex, body mass index and smoking status.

## Data Availability

The original contributions presented in this study are included in the article/Supplementary Materials. Further inquiries can be directed to the corresponding author.

## Supplementary Materials

The following supporting information can be downloaded at https://www.mdpi.com/article/10.3390/ijms262110584/s1.

## Abbreviations

BDNF	Brain-Derived Neurotrophic Factor
COPD	Chronic obstructive pulmonary disease
EGF	Epidermal Growth Factor
FGF	Fibroblast Growth Factor
HGF	Hepatocyte Growth Factor
LIF	Leukemia Inhibitory Factor
NGF-β	Nerve Growth Factor-beta
PDGF	Platelet-Derived Growth Factor
PLGF	Placental Growth Factor
SCF	Stem Cell Factor
VEGF	Vascular Endothelial Growth Factor
