# Supplementary material for: Association Between Serum Growth Factors and Risk of Acute Exacerbation in Chronic Obstructive Pulmonary Disease: A One-Year Prospective Study
Source: Int J Mol Sci. 2025 Oct 30;26(21):10584. doi: 10.3390/ijms262110584 (PMC12610264; doi:10.3390/ijms262110584)
Supplement: Supplementary file 1 [file ijms-26-10584-s001.zip › ijms-3870515-supplementary.pdf]

Supplemental Table S1. Detailed baseline characteristics of the study participants with chronic obstructive pulmonary disease stratified by the presence of acute exacerbation during the 1-year follow up.

Variable	Total (*N* = 112)	Non-AE (*n* = 70)	AE (*n* = 42)	*P*
Demographics				
Male, n (%)	110 (98.2)	69 (98.6)	41 (97.6)	1.000
Age, years	69 (63, 75)	69 (62, 74)	69 (64, 75)	0.371
BMI, kg/m^2	23.6 (21.6, 25.7)	23.7 (22.3, 25.7)	22.4 (20.9, 25.6)	0.115
Smoking, n (%)				1.000
Never	5 (4.5)	3 (4.3)	2 (4.8)	
Current	45 (40.2)	28 (40.0)	17 (40.5)	
Quit	62 (55.4)	39 (55.7)	23 (54.8)	
GOLD classification, n (%)				0.143
1	13 (11.6)	11 (15.7)	2 (4.8)	
2	53 (47.3)	35 (50.0)	18 (42.9)	
3	39 (34.8)	21 (30.0)	18 (42.9)	
4	7 (6.3)	3 (4.3)	4 (9.5)	
GROUP classification, n (%)				0.166
A	46 (41.1)	34 (48.6)	12 (28.6)	
B	23 (20.5)	14 (20.0)	9 (21.4)	
C	25 (22.3)	13 (18.6)	12 (28.6)	
D	18 (16.1)	9 (12.9)	9 (21.4)	
Lung function				
FVC, L	2.6 (2.0, 3.3)	2.9 (2.2, 3.3)	2.3 (1.9, 3.0)	0.006
FVC predicted, %	68 (55, 80)	70 (60, 89)	65 (48, 73)	0.031
FEV1, L	1.3 (1.1, 1.7)	1.4 (1.2, 1.8)	1.2 (0.8, 1.6)	0.010
FEV1 predicted, %	55 (43, 69)	57 (46, 72)	49 (36, 58)	0.004
FEV1 / FVC, %	53 (44, 61)	55 (44, 62)	51 (42, 61)	0.298
DL_{CO}				
DL, ml/min/mmHg	7.2 (4.0, 14.5)	10.3 (4.6, 15.7)	5.1 (2.0, 11.9)	0.008
DL predicted, %	69 (59, 86)	69 (60, 88)	67 (45, 75)	0.118
VA, L	4.4 (3.7, 5.0)	4.6 (3.9, 5.1)	3.9 (3.4, 4.8)	0.012
DL/VA, ml/min/mmHg/L	1.85 (0.89, 3.13)	2.33 (1.06, 3.24)	1.05 (0.53, 2.44)	0.015
DL/VA predicted, %	74 (58, 91)	74 (62, 91)	74 (48, 93)	0.522
Comorbidity				
Cardiovascular disease	9 (8.0)	6 (8.6)	3 (7.1)	1.000
Cerebrovascular disease	19 (17.0)	11 (15.7)	8 (19.0)	0.795
Peptic ulcer disease	19 (17.0)	13 (18.6)	6 (14.3)	0.613
Liver disease	5 (4.5)	4 (5.7)	1 (2.4)	0.649
Diabetes	13 (11.6)	9 (12.9)	4 (9.5)	0.764
Renal disease	5 (4.5)	4 (5.7)	1 (2.4)	0.649
Malignancy	8 (7.1)	3 (4.3)	5 (11.9)	0.149
CCI total score (excluding pulmonary disease)	1.0 (1.0, 2.0)	1.0 (1.0, 2.0)	1.0 (1.0, 3.0)	0.801
Biochemistry data				
White blood count, ×10^3	7.1 (6.0, 8.3)	6.8 (5.6, 8.3)	7.3 (6.3, 7.8)	0.183
Neutrophils, %	60 (55, 65)	59 (54, 64)	62 (59, 68)	0.007
Lymphocytes, %	30 (25, 35)	31 (27, 37)	28 (22, 31)	0.013
Monocytes, %	5.8 (5.2, 7.0)	5.9 (5.2, 7.1)	5.7 (5.1, 6.3)	0.322
Eosinophils, %	3.1 (1.9, 5.0)	3.2 (2.0, 5.4)	3.0 (1.3, 4.5)	0.110
Basophils, %	0.4 (0.2, 0.6)	0.4 (0.3, 0.6)	0.3 (0.2, 0.5)	0.058

Variable	Total (*N* = 112)	Non-AE (*n* = 70)	AE (*n* = 42)	*P*
Fasting glucose, mg/dL	98 (90, 111)	98 (90, 110)	99 (91, 112)	0.947
HbA1C, %	5.8 (5.6, 6.2)	5.8 (5.6, 6.1)	5.9 (5.6, 6.4)	0.585
Triglyceride, mg/dL	111 (81, 158)	121 (87, 166)	88 (71, 135)	0.018
Total cholesterol, mg/dL	192 (157, 208)	194 (163, 211)	180 (150, 206)	0.425
LDL-cholesterol, mg/dL	108 (89, 134)	112 (91, 132)	103 (80, 142)	0.625
HDL-cholesterol, mg/dL	50.0 (42.0, 62.0)	49.0 (41.0, 61.0)	50.0 (44.0, 65.0)	0.360
Urine acid, mg/dL	6.3 (5.4, 7.6)	6.4 (5.4, 7.5)	6.1 (5.4, 8.0)	0.762
BUN, mg/dL	16 (13, 20)	16 (13, 21)	16 (13, 18)	0.640
Creatinine, mg/dL	1.00 (0.90, 1.14)	1.00 (0.90, 1.19)	1.00 (0.89, 1.10)	0.510
SGOT, U/L	20 (16, 26)	21 (17, 26)	18 (16, 26)	0.189
CRP, mg/dL	0.40 (0.18, 0.44)	0.36 (0.18, 0.40)	0.40 (0.16, 0.53)	0.570
Albumin, g/dL	4.5 (4.2, 4.5)	4.5 (4.3, 4.5)	4.3 (4.2, 4.5)	0.093

FVC, forced vital capacity; FEV1, forced expiratory volume in one second; DLCO, diffusing capacity of lung with carbon monoxide; DL, diffusing capacity of lung; VA, alveolar volume; CCI, Charlson Comorbidity Index score; LDL, low density lipoprotein; HDL, high density lipoprotein; SGOT, serum glutamic-oxalocetic transaminase; CRP, C-reactive protein; Data were presented as frequency with percentage or median (interquartile range).

Supplemental Table S2. Baseline characteristics of the study participants with chronic obstructive pulmonary disease stratified by the frequency of acute exacerbation during the 1-year follow up.

Variable	Non-AE (*n* = 70)	AE (*n* = 34)	FAE (*n* = 8)	*P*
Demographics				
Male, n (%)	69 (98.6)	33 (97.1)	8 (100.0)	0.611
Age, years	69 (62, 74)	69 (66, 75)	67 (63, 75)	0.579
BMI, kg/m^2	23.7 (22.3, 25.7)	22.2 (19.3, 24.7)	25.0 (21.7, 26.5)	0.144
Smoking, n (%)				0.989
Never	3 (4.3)	2 (5.9)	0 (0.0)	
Current	28 (40.0)	14 (41.2)	3 (37.5)	
Quit	39 (55.7)	18 (52.9)	5 (62.5)	
GOLD classification, n (%)				0.310
1	11 (15.7)	2 (5.9)	0 (0.0)	
2	35 (50.0)	13 (38.2)	5 (62.5)	
3	21 (30.0)	15 (44.1)	3 (37.5)	
4	3 (4.3)	4 (11.8)	0 (0.0)	
GROUP classification, n (%)				0.299
A	34 (48.6)	10 (29.4)	2 (25.0)	
B	14 (20.0)	6 (17.6)	3 (37.5)	
C	13 (18.6)	10 (29.4)	2 (25.0)	
D	9 (12.9)	8 (23.5)	1 (12.5)	
Lung function				
FVC, L	2.9 (2.2, 3.3)	2.2 (1.8, 2.7)	2.6 (2.3, 3.3)	0.009
FVC predicted, %	70 (60, 89)	65 (48, 72)	69 (53, 80)	0.084
FEV1, L	1.4 (1.2, 1.8)	1.2 (0.8, 1.4)	1.5 (1.1, 1.9)	0.010
FEV1 predicted, %	57 (46, 72)	49 (36, 58)	56 (37, 61)	0.014
FEV1 / FVC, %	55 (44, 62)	54 (41, 61)	49 (44, 59)	0.581
DLCO				
DL, ml/min/mmHg	10.3 (4.6, 15.7)	5.5 (2.0, 9.8)	4.2 (1.5, 15.5)	0.030
DL predicted, %	69 (60, 88)	62 (45, 69)	74 (44, 87)	0.197
VA, L	4.6 (3.9, 5.1)	3.8 (3.4, 4.8)	4.2 (3.5, 4.7)	0.041
DL/VA, ml/min/mmHg/L	2.3 (1.1, 3.2)	1.4 (0.6, 2.4)	0.8 (0.4, 3.7)	0.051
DL/VA predicted, %	74 (62, 91)	63 (48, 85)	85 (47, 102)	0.523
Comorbidity				
Cardiovascular disease	6 (8.6)	2 (5.9)	1 (12.5)	0.735
Cerebrovascular disease	11 (15.7)	5 (14.7)	3 (37.5)	0.304
Peptic ulcer disease	13 (18.6)	5 (14.7)	1 (12.5)	0.918
Liver disease	4 (5.7)	1 (2.9)	0 (0.0)	1.000
Diabetes	9 (12.9)	3 (8.8)	1 (12.5)	0.897
Renal disease	4 (5.7)	1 (2.9)	0 (0.0)	1.000
Malignancy	3 (4.3)	5 (14.7)	0 (0.0)	0.194
CCI total score (excluding pulmonary disease)	1.0 (1.0, 2.0)	1.0 (1.0, 3.0)	2.0 (1.0, 2.0)	0.965
Biochemistry data				
White blood count, ×10^3	6.8 (5.6, 8.3)	7.1 (6.3, 7.8)	7.8 (7.5, 8.3)	0.132
Neutrophils, %	59 (54, 64)	62 (59, 68)	61 (56, 67)	0.020
Lymphocytes, %	31 (27, 37)	28 (22, 31)	28 (22, 32)	0.046
Monocytes, %	5.9 (5.2, 7.1)	5.8 (5.1, 6.3)	5.5 (5.2, 6.2)	0.563

Eosinophils, %	3.2 (2.0, 5.4)	2.6 (1.3, 4.1)	4.0 (1.8, 6.6)	0.128
Basophils, %	0.40 (0.30)	0.30 (0.30)	0.40 (0.35)	0.081
Fasting glucose, mg/dL	98 (90, 110)	97 (91, 113)	103 (86, 110)	0.987
HbA1C, %	5.8 (5.6, 6.1)	5.9 (5.5, 6.3)	6.1 (5.9, 7.0)	0.187
Triglyceride, mg/dL	121 (87, 166)	85 (71, 128)	123 (81, 149)	0.032
Total cholesterol, mg/dL	194 (163, 211)	180 (149, 206)	179 (151, 227)	0.672
LDL-cholesterol, mg/dL	112 (91, 132)	104 (85, 142)	89 (78, 144)	0.765
HDL-cholesterol, mg/dL	49 (41, 61)	50 (43, 66)	53 (46, 59)	0.652
Urine acid, mg/dL	6.4 (5.4, 7.5)	6.3 (5.6, 8.1)	5.8 (5.2, 7.9)	0.895
BUN, mg/dL	16 (13, 21)	16 (13, 17)	14 (14, 37)	0.825
Creatinine, mg/dL	1.00 (0.29)	1.00 (0.20)	1.00 (0.53)	0.792
SGOT, U/L	21 (17, 26)	19 (16, 26)	16 (14, 24)	0.221
CRP, mg/dL	0.36 (0.22)	0.40 (0.43)	0.39 (0.22)	0.841
Albumin, g/dL	4.5 (4.3, 4.5)	4.4 (4.2, 4.5)	4.2 (4.2, 4.6)	0.216

FVC, forced vital capacity; FEV1, forced expiratory volume in one second; DLCO, diffusing capacity of lung with carbon monoxide; DL, diffusing capacity of lung; VA, alveolar volume; CCI, Charlson Comorbidity Index score; LDL, low density lipoprotein; HDL, high density lipoprotein; SGOT, serum glutamic-oxalocetic transaminase; CRP, C-reactive protein; Data were presented as frequency with percentage or median (interquartile range).

Supplemental Table S3. The relationship between blood eosinophil count and the level of the tested 11 growth factors

Growth factor	Spearman's rank correlation	*P* value
NGF	-0.02	0.878
BDNF	-0.03	0.756
EGF	-0.08	0.431
FGF-2	0.04	0.641
HGF	-0.08	0.390
LIF	0.05	0.602
PDGF	-0.09	0.354
PLGF	0.01	0.883
SCF	-0.01	0.929
VEGF-A	-0.01	0.904
VEGF-D	-0.08	0.422

Abbreviation: NGF, nerve growth factor; BDNF, brain-derived neurotrophic factor; EGF, epidermal growth factor; FGF-2, fibroblast growth Factor-2; HGF, hepatocyte growth factor; LIF, leukemia inhibitory factor; PDGF, platelet-derived growth factor; PLGF, placental growth factor; SCF, stem cell factor; VEGF-A, vascular endothelial growth factor-A; VEGF-D, vascular endothelial growth factor-D.

Supplemental Table S4. The value of growth markers according to the frequency of acute exacerbation during the 1-year follow up.

**Variable, pg/ml**	**Non-AE (*n* = 70)**	**AE (*n* = 34)**	**FAE (*n* = 8)**	***P*§**
NGF	32.5 (16.1, 61.4)	29.2 (14.2, 58.0)	3.7 (0.7, 20.4)	0.018
BDNF	97.0 (65.7, 174.5)	98.5 (60.5, 128.2)	88.6 (51.8, 154.7)	0.813
EGF	29.5 (19.0, 47.6)	27.4 (18.6, 42.6)	17.1 (14.3, 22.8)	0.053
FGF-2	12.4 (0.8, 30.0)	5.0 (0.0, 20.4)	0.1 (0.0, 4.3)	0.030
HGF	172.9 (151.4, 244.1)	201.8 (155.1, 242.5)	241.4 (174.6, 298.4)	0.323
LIF	3.1 (2.4, 4.7)	2.7 (2.3, 3.6)	1.9 (1.6, 3.1)	0.061
PDGF	255.0 (164.8, 431.3)	273.7 (199.6, 378.2)	235.0 (116.6, 359.7)	0.788
PLGF	11.0 (6.4, 18.8)	9.7 (6.5, 14.5)	7.8 (3.6, 19.2)	0.622
SCF	16.9 (13.0, 25.0)	18.0 (14.9, 32.5)	18.9 (12.1, 28.8)	0.279
VEGF-A	82.8 (53.6, 136.2)	85.1 (63.5, 125.6)	52.8 (40.0, 108.2)	0.298
VEGF-D	8.1 (5.7, 14.7)	9.4 (4.2, 13.6)	5.9 (4.1, 9.6)	0.374

Abbreviation: AE, acute exacerbation; NGF, nerve growth factor; BDNF, brain-derived neurotrophic factor; EGF, epidermal growth factor; FGF-2, fibroblast growth Factor-2; HGF, hepatocyte growth factor; LIF, leukemia inhibitory factor; PDGF, platelet-derived growth factor; PLGF, placental growth factor; SCF, stem cell factor; VEGF-A, vascular endothelial growth factor-A; VEGF-D, vascular endothelial growth factor-D; Data were presented as median (interquartile range); § Kruskal-Wallis test.
