# Supplementary figures and images for: Association Between Serum Growth Factors and Risk of Acute Exacerbation in Chronic Obstructive Pulmonary Disease: A One-Year Prospective Study
Source: Int J Mol Sci. 2025 Oct 30;26(21):10584. doi: 10.3390/ijms262110584 (PMC12610264; doi:10.3390/ijms262110584)

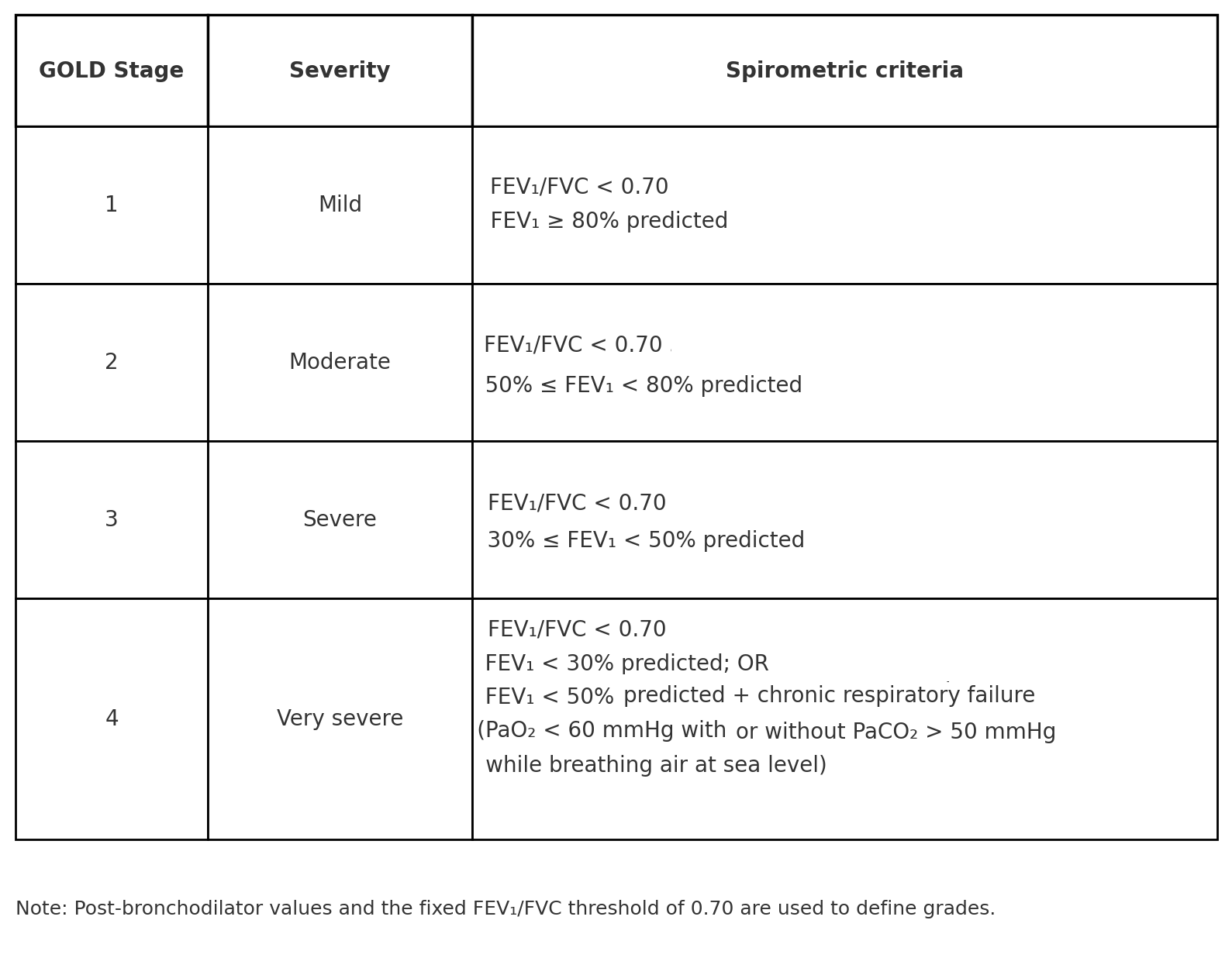

Supplement: Supplementary file 1 [file ijms-26-10584-s001.zip › ijms-3870515-supplementary.png]
